# Supplementary material for: Goose Hepatic IGFBP2 Is Regulated by Nutritional Status and Participates in Energy Metabolism Mainly through the Cytokine−Cytokine Receptor Pathway
Source: Animals (Basel). 2023 Jul 17;13(14):2336. doi: 10.3390/ani13142336 (PMC10376900; doi:10.3390/ani13142336)
Supplement: Supplementary file 1 [file animals-13-02336-s001.zip › animals-2444240-supplementary/Supplementary Files/Supplementary materials 20230717.pdf]

Supplementary Figure S1

(A)

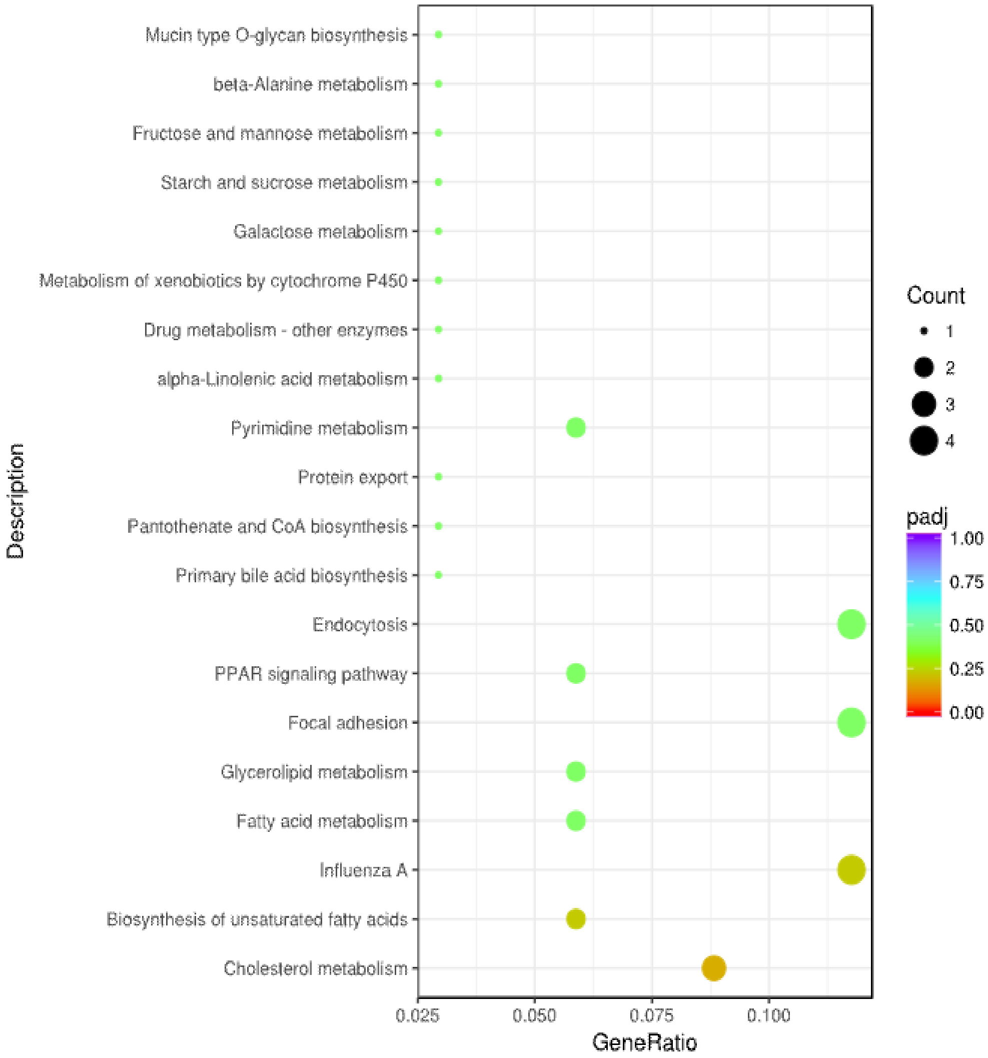

(B)

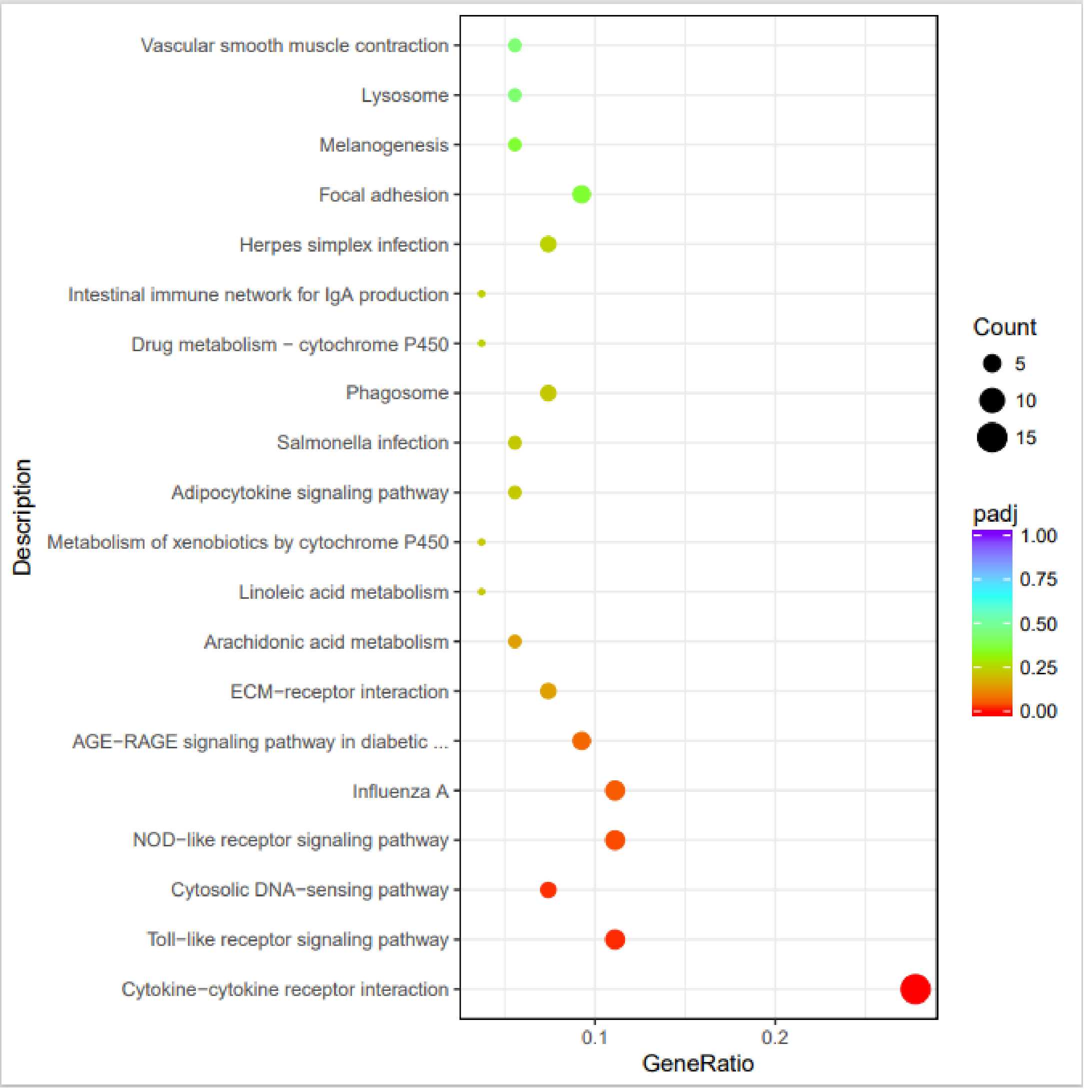

Supplementary Figure S1. The dot chart showing the results from KEGG enrichment analysis. Note: (A) The results of KEGG enrichment analysis with the upregulated DEGs. (B) The results of KEGG enrichment analysis with the downregulated DEGs. The abscissa indicates the ratio of the number of the annotated DEGs in a specified KEGG pathway to the total number of annotated DEGs, and the ordinate indicates the KEGG pathways. The colors denote the adjusted *p* value (padj), and the sizes of the dots denote the number of DEGs. The DEGs were identified in goose primary hepatocytes transfected with IGFBP2 overexpression vectors vs. empty vectors (n = 4).

Supplementary Table S1. The sequences of the primers for qPCR analysis

Gene	Sequence (5'→3')	Product Size (bp)	Accession number
IGFBP2	F: GGGAGAAGGTGAATGAGCAG<br>R: GAGGGAGTAGAGGTGCTCCA	194	XM_013196707.2
MAPCK7CL	F: CAGCCTCTGCCTCCTTGTC<br>R: GCTTCAGCGTTCGGTTCTC	226	XM_048071423.1
SLC40A1	F: CTGGGGAGATCGTATGTGGC<br>R: AGGATGTCTGGGCCACTTTG	179	XM_048058484.1
STAT4	F: GCACGCCTTTTGTTCGGTAT<br>R: TCCATTGGGACATACTCAGCAC	200	XM_048058203.1
LOC106041089	F: CCCAGCCAGGCGTGATATTC<br>R: TGATGCTTGAAGGGAGCAGG	143	XM_013189339.2
IL6	F:CGACGATAAGGCAGATGGTGATA<br>R: ACAGCCCTCACGGTTTTCTC	172	XM_048070285.1
LOC106037025	F: CAGAAGACAGGAGGTGAGCC<br>R: GTCATCCTGTGCCTCCTATGG	115	XM_013182731.2
LOC106041041	F: CAGCTTCGTGACCGACTACT<br>R: CCTTCCTTGTGATGAACACGAC	154	XM_013189266.2
IL22RA1	F: TCAACCAACGGACACACCAA<br>R: AAGCCATGTGGTGTCTGCTA	173	XM_013184113.2
LOC106041919	F: CAGAGAAGTGTGCCTGGACC<br>R: GCTTGGTGTTTGCCTTGTCC	80	XM_013190619.2
LOC106038554	F: ACGAGCAATGTGACCGTCTT<br>R: ACCCAGTGTTTTACCCAGCC	238	XM_048070544.1
LOC106041040	F: GCTGACACAACCATCTGCTG<br>R: ACCTCCTGGTGATGAACACAACT	123	XM_013189265.2
LOC101042256	F: TGCAAACTGCGGAAGATAATGA<br>R: GGCAAGGGAAATGAATCCAGC	210	XM_013191039.2
CCL20	F: AGTACCTCCCAAGCACAAAGC<br>R: CGCAGACTTCACTGGAGAGC	112	XM_013181430.2
RSAD2	F: GCGTCAACTACCACTTCACC<br>R: AATTTTCTCCATTCCCGCCTCC	140	XM_013172803.2
FGB	F: ACCCAAACAGACTACTGCCG<br>R: TGAATCAAAGTCCAGCCTCCA	197	XM_013175323.2
CD9	F: TTTAAGGAGTGGCGCTGTGG<br>R: TTAGTGCCTCCTTTGACGGG	108	XM_013196002.2
COTL1	F: GAGGTGTCACGGCACAGTAA<br>R: CTCCTGAGCCCCAGCATTAG	117	XM_013195033.2
Β-ACTIN	F: CAACGAGCGGTTCAGGTGT<br>R: TGGAGTTGAAGGTGGTCTCG	132	XM_013174886.1
GAPDH	F: GCCATCAATGATCCCTTCAT<br>R: CTGGGGTCACGCTCCTG	155	XM_013199522.2

Supplementary Table S2. The data volume and mapping rate for RNAseq

Sample ID	Data volume (base pair)	Mapping rate (%)
C1	7.7G	72.9
C2	7.0G	69.7
C3	7.9G	71.5
C4	6.3G	72.4
T1	7.0G	66.7
T2	6.7G	73.0
T3	7.2G	73.1
T4	7.0G	73.6

Supplementary Table S3. Top 10 up- and down-regulated differentially expressed genes (sorted by fold change)

Gene name	Gene symbol	Log_2(Fold Change)	*p*-Value	adjusted *p*-value
Upregulated				
Solute Carrier Family 34 Member 2	*SLC34A2*	1.84	4.08×10^{-7}	4.99×10^{-5}
Diras Family Gtpase 2	*DIRAS2*	1.71	5.05×10^{-4}	2.10×10^{-2}
Adhesion G Protein-coupled Receptor D1	*ADGRD1*	1.39	1.91×10^{-4}	5.96×10^{-3}
Goosecoid Homeobox	*GSC*	1.37	1.04×10^{-4}	1.80×10^{-3}
Collectin Subfamily Member 10	*COLEC10*	1.34	2.64×10^{-5}	$1.50 \times 10^{-}$
Uncharacterized LOC106032316	*LOC106032316*	1.32	7.46×10^{-10}	9.30×10^{-3}
MAP3K7 C-terminal Like	*MAP3K7CL*	1.30	1.84×10^{-4}	1.11×10^{-4}
Matrix Gla Protein	*MGP*	1.29	1.03×10^{-6}	2.91×10^{-6}
Riboflavin-Binding Protein	*LOC106033105*	1.17	1.86×10^{-8}	2.35×10^{-3}
Keratin, Type I Cytoskeletal 19	*LOC106048013*	1.17	3.42×10^{-5}	1.36×10^{-4}
Downregulated				
Interleukin 17A	*IL17A*	-2.77	6.05×10^{-12}	1.84×10^{-9}
Src Family Transcript Variant X1	*BLK*	-2.15	6.77×10^{-6}	5.74×10^{-4}
Toll-Like Receptor 2	*LOC106042256*	-2.07	4.86×10^{-28}	7.81×10^{-25}
Ovostatin-Like	*LOC106048083*	-1.99	3.38×10^{-10}	7.06×10^{-8}
Cysteine-Rich Secretory Protein LCCL Domain Containing 2	*CRISPLD2*	-1.89	5.19×10^{-5}	3.40×10^{-3}
Serpin B4-Like	*LOC106048987*	-1.77	1.30×10^{-4}	7.13×10^{-3}
ADAM Metallopeptidase With Thrombospondin Type 1 Motif 15	*ADAMTS15*	-1.76	1.29×10^{-4}	7.12×10^{-3}
Family With Sequence Similarity 167 Member A	*FAM167A*	-1.73	2.39×10^{-11}	6.41×10^{-9}
C-C Motif Chemokine Ligand 20	*CCL20*	-1.68	2.46×10^{-8}	3.74×10^{-6}
Retinoic Acid-Induced Protein 3-Like	*LOC106045506*	-1.65	1.50×10^{-48}	1.30×10^{-2}

Note: The differentially expressed genes (DEGs) were identified from transcriptome analysis of goose primary hepatocytes transfected with IGFBP2 overexpression vector and empty vector (the control). The genes shown in this table are the top 10 upregulated or downregulated DEGs with the largest |log2(fold change)| values. The fold change refers to the ratio of expression level of the DEG in the IGFBP2-overexpressing cells to that of the control cells. The letter 'E' indicates the scientific notation.

Supplementary Table S4. The KEGG pathways significantly enriched with the differentially expressed genes (DEGs)

KEGG pathways	The differentially expressed genes
**Cytokine-cytokine receptor interaction**	LOC106041040/IL6/IL2RA/IL22RA1/LOC106038123/LOC106041041/IL17A/SSF49265/CCL20/LIF/LOC106038554/LOC106041919/IL22RA2/FIGF/TNFRSF1B/LOC106048600
**Cytosolic DNA-sensing pathway**	LOC106041040/IL6/LOC106041041/NFKBIA
**Toll-like receptor signaling pathway**	LOC106041040/IL6/LOC106041041/NFKBIA/LOC106041919/PIK3R3
**AGE-RAGE signaling pathway in diabetic complications**	IL6/LOC106041602/LOC106041919/FIGF/PIK3R3/PLCB2
**Arachidonic acid metabolism**	PTGS2/HPGDS/LOC106033102/PTGES
**Metabolism of xenobiotics by cytochrome P450**	HPGDS/MGST2/ALDH1A3
**Focal adhesion**	VVF/LOC106038554/CAV2/LAMB3/FIGF/ITGB5/CAV1/PIK3R3/ITGB4
**NOD-like receptor signaling pathway**	LOC106041040/IL6/NFKBIA/LOC106041919/ANTXR2/PLCB2
